# Unlocking expertise: the application of threshold concepts in scenario simulations in undergraduate clinical teaching

**DOI:** 10.3389/fmed.2025.1690297

**Published:** 2025-10-17

**Authors:** Dandan Ma, Ning Su, Rui Hou, Yanyan Wan, Zhuoran He, Jinzhong Lan, Guizhen Xiao, Mian Peng

**Affiliations:** ^1^Department of Critical Care Medicine, Luohu Clinical Institute of Shantou University Medical College (Shenzhen Luohu People’s Hospital), Shenzhen, China; ^2^Department of Science and Education, Luohu Clinical Institute of Shantou University Medical College (Shenzhen Luohu People’s Hospital), Shenzhen, China; ^3^Department of Critical Care Medicine, The Third Affiliated Hospital of Shenzhen University, Shenzhen, China; ^4^Department of Clinical Nutrition, Southern University of Science and Technology Hospital, Shenzhen, China

**Keywords:** threshold concepts, medical education, scenario-based simulation, clinical skills, teaching strategies

## Abstract

**Background:**

Threshold concepts, as a transformative learning approach, are crucial for mastering new knowledge and skills. They help students change their understanding of subjects and promote the development of professional competencies. This study aims to identify and understand the key threshold concepts in undergraduate clinical medical education and to explore their effectiveness in scenario-based simulation teaching.

**Methods:**

Using a comparative experimental design, students were divided into two groups: one receiving threshold concept training and the other receiving traditional reinforcement training. The study combined scenario-based simulation, semi-structured interviews, and open-text surveys to assess student performance before and after training.

**Results:**

Students who received threshold concept training performed significantly better in tests than those in the traditional methods group. In Case 1, the threshold concept group scored 251(203, 259), significantly higher than the traditional methods group’s 201(181, 249) (*p* < 0.05). In Case 2, the threshold concept group scored 245(236, 251), significantly higher than the traditional methods group’s 232(228, 237) (*p* < 0.05). The results indicate that threshold concept training significantly improves student test scores, with statistically significant differences.

**Conclusion:**

This study identified key threshold concepts in undergraduate clinical education and validated their effectiveness in scenario-based simulation teaching. Threshold concepts not only guide the development of disease diagnosis and treatment skills but also provide new directions for teaching design and learning activities, facilitating the transition of medical students into competent doctors.

## Background

Medical education, as a core component of training future clinicians, consistently faces the challenge of effectively bridging theoretical knowledge with clinical practice. Traditional clinical teaching emphasizes knowledge transmission and skill training, but students often struggle with the integration and application of knowledge in complex clinical scenarios. Particularly at the undergraduate level, medical students need to navigate the critical threshold from ‘basic cognition’ to ‘professional practice.’ This process is frequently hampered by the conceptual barriers posed by complex or abstract concepts, forming ‘learning bottlenecks’ (1). Innovative educational strategies are demonstrating significant potential to help students cross this threshold. For instance, combining flipped classroom with simulation-based training has been shown to markedly boost medical students’ self-perceived clinical competence and confidence (2). Similarly, exploring cost-effective alternatives, such as PC-based tools, can make simulation training equally effective and more accessible, thereby further facilitating this essential transition in skill acquisition (3). Against this backdrop, the theoretical framework of threshold concepts offers a new perspective to address this issue.

Threshold concepts, first introduced by Meyer and Land (4), refer to core concepts within a discipline that possess transformative, irreversible, and integrative qualities. Once mastered, these concepts fundamentally alter the learner’s understanding of the subject and serve as gateways to further learning. In the field of medicine, such concepts may encompass pathophysiological mechanisms (e.g., acid–base balance, immune response), clinical decision-making logic (e.g., Bayesian reasoning in diagnostic reasoning), or ethical practice principles (e.g., the balance between patient autonomy and medical intervention) (5). However, these concepts often present high cognitive thresholds. If students fail to overcome these barriers, they may remain in a state of ‘partial understanding,’ impeding the development of clinical competence.

Simultaneously, scenario-based simulation, as a practice-oriented teaching method, has been widely applied in medical education in recent years (6). By providing highly realistic clinical scenarios, it allows students to experience real medical situations in a safe environment, promoting knowledge transfer and the cultivation of critical thinking. Nevertheless, existing research predominantly focuses on the training of operational skills and team collaboration, with limited attention to the design of scenario-based simulations that precisely target the ‘cognitive breakthrough points’ of threshold concepts, thereby systematically addressing students’ learning barriers (7).

In our previous study (8), we implemented a comprehensive emergency simulation training for clinical medical students (second to fourth-year undergraduates). The results indicated that timely feedback, repeated practice, curriculum integration, and the use of realism-based simulations can enhance students’ ability to manage patients in clinical emergencies. However, to ensure greater success in interdisciplinary simulation training for undergraduate clinical medical students, it is crucial to decode the core concepts in teaching and learning.

To help students overcome challenges in learning a subject, this study focuses on the intersection of ‘threshold concepts’ and ‘scenario-based simulation,’ exploring how structured, progressive simulation teaching designs can help medical students cross critical thresholds in professional knowledge (1). Therefore, this study developed a context-integrated simulation training course, aiming to identify and understand these threshold concepts that are essential for undergraduates to master medical knowledge. This direction not only aligns with the trend of shifting medical education from ‘knowledge transmission’ to ‘competency-oriented’ but also provides theoretical foundations and practical pathways for optimizing clinical teaching models and enhancing students’ clinical thinking and decision-making abilities.

## Methods

### Design of the study

This study employed a mixed research method, utilizing semi-structured interviews and open-text surveys. The research team initially conducted in-depth interviews with clinical teachers, focusing on four core dimensions: clinical thinking, skill operations, humanistic spirit, and teamwork. Concurrently, open-ended free-text questionnaires were administered to medical students. Following the data collection phase, the research team organized focus group discussions with expert teachers, concentrating on the cognitive barriers and developmental bottlenecks that medical students encounter while mastering clinical threshold concepts. This process systematically identified the key obstructive factors affecting the cultivation of clinical competencies.

### Participants

Full-time Grade 4 clinical medicine students from Shantou University Medical College were eligible for this study. Among the 35 students in the Shenzhen class, 30 were enrolled after excluding those who had previously received first aid training. Participants were randomly assigned to either the Threshold Concept group or the Traditional Reinforcement group using a computer-generated random number sequence. The study spanned 2 months, from September 1 to October 30, 2023. This preliminary exploratory study utilized a convenience sample of all available students from a single cohort to primarily assess the feasibility and potential effect size of the intervention.

### Study procedure

The goal of contextual simulation training for 4th-year undergraduate clinical medicine students is to develop their clinical thinking, practical skills, humanism, and teamwork in an interdisciplinary context. The mannequins used for first aid training are realistic, anatomically accurate, computer-driven, and capable of simulating the physiological responses of real patients. Each simulated first aid session lasts 2 h, with 15 students per group and a student-teacher ratio of 5:1. Two sets of interdisciplinary situational simulation training programs were designed, validated by authoritative experts for consistency in type and difficulty. These two training methods were applied cross-sectionally. In both methods, students receive regular interdisciplinary simulation training in the first month and intensive training in the second month, allowing them a period for cognitive assimilation with the simulation program and providing a foundational basis for threshold concepts.

Case 1: A 35-year-old female patient presented with recurrent fever, cough, expectoration, and joint swelling and pain for over 10 days. After receiving antibiotic treatment at a local hospital, she developed respiratory failure and was transferred to our Emergency Intensive Care Unit today.

Case 2: A 35-year-old male patient, after heavy drinking, engaged in a physical altercation and sustained multiple kicks to the abdomen. Three hours later, he presented to our emergency department with severe abdominal pain, accompanied by dizziness, fatigue, and sweating.

#### Assessment tools

Student performance was evaluated using a standardized, validated checklist rubric for each simulation case. The rubrics assessed critical domains including history-taking, physical examination, clinical reasoning, diagnosis, and treatment planning.

Part 1: Initial Patient Assessment (Total score: 100 points): Medical History Taking: 35 points; Physical Examination: 22 points; Preliminary Diagnosis: 5 points; Initial Treatment: 28 points; Physician-Patient Communication: 10 points.

Part 2: Diagnostic and Therapeutic Procedures (Total score: 100 points).

Case 1: Endotracheal Intubation: 80 points; Balloon-Assisted Ventilation: 20 points; Case 2: Diagnostic Abdominal Paracentesis: 80 points; Pre-Procedural Assessment: 20 points.

Part 3: Management of Clinical Deterioration (Total score: 100 points).

Case 1: Thoracentesis for Pneumothorax: 80 points; Physician-Patient Communication: 20 points; Case 2: Management of Hemorrhagic Shock: Pre-Shock Assessment, Shock Correction, and Surgical Intervention: 80 points; Physician-Patient Communication: 20 points.

#### Training method

**Figure 1:**
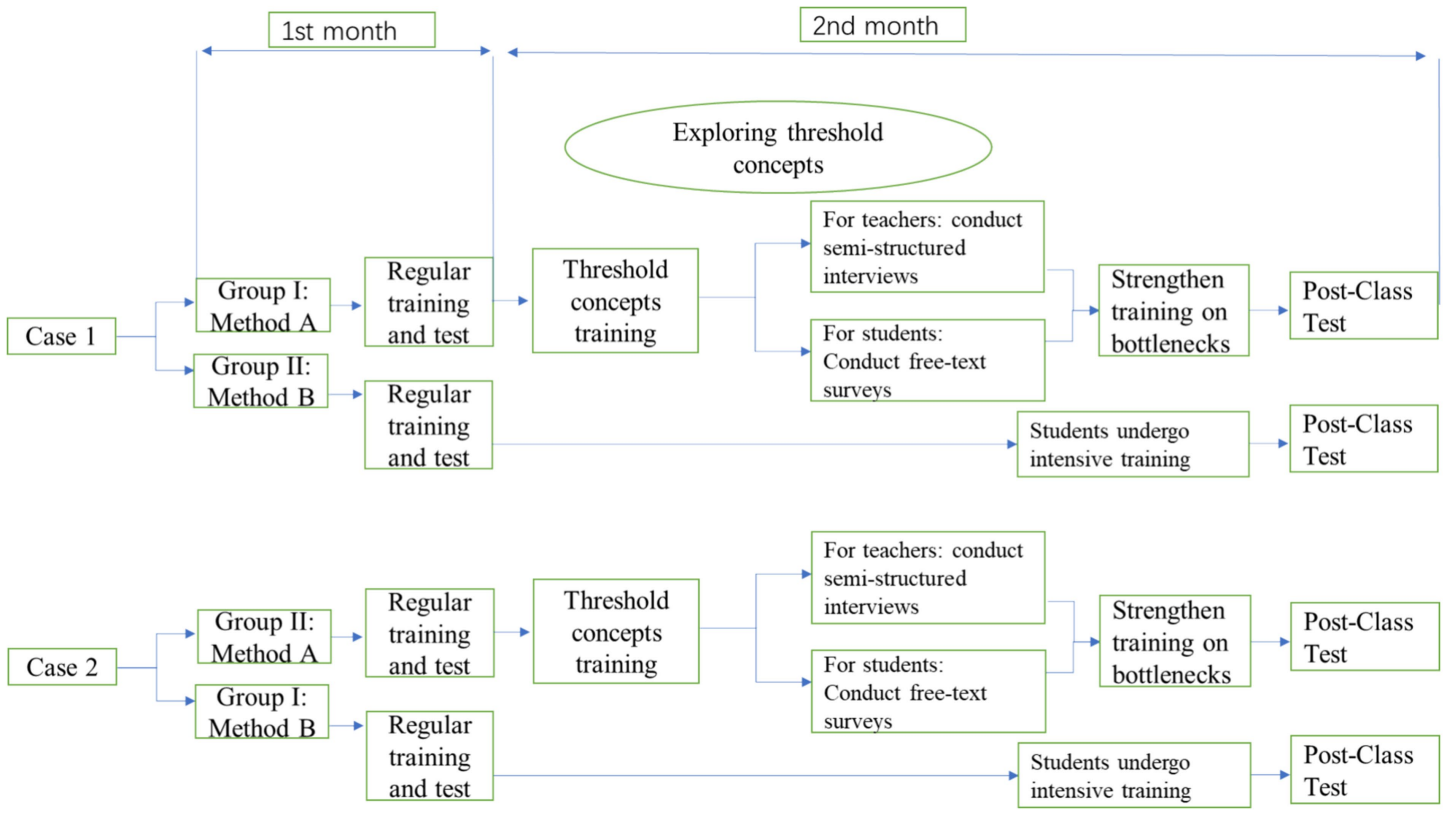
The general flowchart of the whole research.

In both training methods, students received regular interdisciplinary simulation training during the first month. In the second month, after students had gained initial insights and a foundational understanding of the interdisciplinary simulation training program, they underwent intensive training. For training method A, students received threshold concepts training before the intensive training in the second month. Subsequently, semi-structured interviews were conducted with teachers, and free-text surveys were administered to medical students. For training method B, the intensive training in the second month remained consistent with the first month’s training. The study procedure was listed in Figure 1.

#### Blinding

The instructors delivering the training were not blinded to group allocation due to the nature of the intervention. However, the faculty assessors who scored the student performances in the simulation scenarios were blinded to the group assignment to minimize assessment bias.

#### Assessors and calibration

Two experienced clinical educators, who were blinded to the group allocation of the students, independently scored all video-recorded simulation performances. Prior to the assessment, both raters participated in a calibration session where they jointly scored sample recordings not included in the study to ensure a consistent understanding and application of the scoring rubric.

### Interviews with educators

A semi-structured, one-to-one, face-to-face interview was conducted with six teachers to identify the threshold concept. As part of the interview process, we informed the participants about threshold concepts beforehand, but explained that their input would not be analyzed. We used open questions proposed by Cousin’s research (9) into threshold concepts, such as possible student misconceptions, specific topics that students had difficulty mastering, and barriers that students encountered in learning these topics. How can we enhance medical students’ diagnostic and therapeutic abilities following a clear diagnosis? How do students demonstrate humanistic care throughout the simulation training for critically ill patients? How should course design be approached in the teaching and learning process?

### Free-text surveys to medical students

What were the most significant challenges encountered during interdisciplinary simulation training? Which part of the course did you find most beneficial, and why? How effective was team collaboration throughout the simulation training? Were you able to accurately perform the necessary skills in response to changes in the condition of critically ill patients? What knowledge do you hope to gain in future training sessions?

### Outcome measures

The primary outcome of this research project was the results of the method A and method B post-course tests. The secondary outcomes were self-efficacy scales and student satisfaction with threshold concept training. The questionnaire was measured on a 5-point Likert scale ranging from 0 (Not at all) to 4 (Totally). Training feedback forms and self-efficacy questionnaires were distributed in paper format after each training session. Students took a post-training test and completed the questionnaires.

### Statistical analysis

All analyzes were performed by using SPSS version 22 (IBM Inc., New York, United States). All statistical tests were 2 sided, and *p*-value < 0.05 indicated statistical significance. An independent t test was performed to compare the continuous variables of the normal distribution, and a Mann–Whitney *U* test was performed to compare the continuous variables of skew distribution. If the data do not follow a normal distribution, express them as median and interquartile range, and use the rank-sum test.

## Results

For the cohort of 30 medical students enrolled in the study, none was excluded or declined to participate. Questionnaire response rates for each group and learning reactionnaire are 100%. According to the Shapiro–Wilk test, the pre- and post-training scores of both groups of students, as well as the differences between them, did not conform to a normal distribution (*p* < 0.05). Comparisons using the Wilcoxon-rank test.

In case 1, group 1 received training via the threshold concept approach, whereas group 2 was trained using the conventional reinforcement method. Prior to training, there was no statistical difference between the two groups (*p* > 0.05, see Table 1). After training, however, group 1’s score was 251(203, 259) while group 2’s score was 201(181, 249). Group 1 scored significantly higher than group 2, and the difference was statistically significant (*p* < 0.05, see Table 2).

**Table 1:** Comparison of pre-training scores for Case 1 and Case 2 between the two student groups: M (P25, P75).

	*n*	Case 1	Case 2
Group 1	15	148(111, 167)	126(118, 179)
Group 2	15	146(116, 152)	119(112, 172)
*Z*		0.643	1.597
*P*		0.539	0.116

**Table 2:** Scores of the two student groups after training with different methods: M (P25, P75).

	Group 1	Group 2	*Z*	*P*
Case 1	Exploring threshold concepts 251(203, 259)	Normal training 201(181, 249)	−2.096	0.037
Case 2	Normal training 232(228, 237)	Exploring threshold concepts 245(236, 251)	−2.577	0.009

In case 2, group 2 received training via the threshold concept approach, whereas group 1 was trained using the conventional reinforcement method. Similarly, there was no statistical difference between the two groups before training (*p* > 0.05, see Table 1). After training, however, group 2’s score was 245(236, 251) while group 1’s score was 232(228, 237). Group 2 scored significantly higher than group 1, and the difference was statistically significant (*p* < 0.05, see Table 2).

In terms of comparing the scores of the two groups of students before and after the training, the scores after the training can be significantly higher than the pre-training scores, but the elevation of the scores after the threshold concept training is more obvious (*p* < 0.001, see Table 3).

**Table 3:** Scores before and after for both groups of students M (P25, P75).

	Teaching method: exploring threshold concepts				*P*
	pre	post	*d*	*Z*	
Group 1 (case 1)	148(111, 167)	251(203, 259)	81(100, 110)	3.408	<0.001
Group 2 (case 2)	119(112, 172)	245(236, 251)	128(58, 137)	3.408	<0.001

	Teaching method: normal				P
	Pre	Post	*d*	*Z*	
Group 1 (case 2)	126(118, 179)	232(228, 237)	67(101, 113)	3.411	<0.001
Group 2 (case 1)	146(116, 152)	201(181, 249)	29(53, 138)	3.408	<0.001

A total of 30 students from two group provided their complete feedback on the threshold concepts training (Table 4). For this course, 96.7% of the students were satisfied with our threshold concepts training and more than 90% have a good evaluation (evaluation of the necessity, 90.0%; evaluation of the form, 96.6%; evaluation of the schedule, 83.3%; level of participation, 93.4%). The majority of these students also thought that threshold concepts training may help them improve themselves effectively (90.0%). Moreover, 86.7% of the students were satisfied with their teacher.

**Table 4:** The feedback of students.

	0 point	1 point	2 point	3 point	4 point
	Very disagree to satisfied				
My overall satisfaction with this exploring threshold concepts course	0.0%	3.3%	0.0%	50.0%	46.7%
Evaluation of the necessity of this exploring threshold concepts course	0.0%	3.3%	6.7%	43.3%	46.7%
Evaluation of the form of this exploring threshold concepts course	0.0%	3.3%	0.0%	53.3%	43.3%
Evaluation of the schedule of this exploring threshold concepts course	0.0%	0.0%	6.7%	40.0%	53.3%
My level of participation in the process of this course	0.0%	3.3%	3.3%	46.7%	46.7%
Exploring threshold concepts course helps me better grasp the clinical operation	0.0%	6.7%	3.3%	43.3%	46.7%
My satisfaction with the teachers of exploring threshold concepts course	3.3%	3.3%	6.7%	30.0%	56.7%

At the end of the training, participants completed a self-efficacy scales. The results showed that, after training in the threshold concept, 80 percent of the students felt that they were able to grasp what they had learned in class in a timely manner and that they understood what they had learned; 73.3% believed they could apply their knowledge, while 83% felt they could relate it to prior learning when addressing problems or reading textbooks (see Table 5).

**Table 5:** Student self-efficacy scales.

	Completely inconsistent	Basically inconsistent	Uncertain	Basically consistent	Completely consistent
1. I am able to comprehend the material presented in class promptly	3.3%	0.0%	16.7%	60.0%	20.0%
2. I can apply what I’ve learned.	0.0%	3.3%	23.3%	53.3%	20.0%
3. I feel confident in my understanding of the knowledge conveyed in textbooks as well as in the instruction provided by my teacher	6.7%	3.3%	10.0%	60.0%	20.0%
4. When considering a specific medical issue, I can integrate it with the medical knowledge I have acquired	0.0%	6.7%	10.0%	43.3%	40.0%
5. While reading medical textbooks, I am able to relate the content to the knowledge I have previously mastered	0.0%	3.3%	13.3%	46.7%	36.7%

## Discussion

Research indicates that threshold concepts can guide educators in designing effective curricula (10). When designing courses, educators need to identify foundational content, pinpoint bottlenecks and challenges in the learning process, and determine which knowledge points require more time for explanation. The introduction of threshold concepts helps teachers organize student learning around these critical “bottleneck” concepts, which can lead to significant shifts in understanding. For instance, ecological balance has been identified as a threshold concept. Consequently, educators allow ample time for students to reflect, research, and discuss, ensuring that students fully comprehend that disciplines like physiology, biochemistry, and clinical diseases often result from disrupted balance.

Threshold concepts represent a new theory that offers educators fresh insights and transforms our approach to problem-solving. In this study, the threshold concept is primarily illustrated through the practical model of clinical teaching (11). Prior to introducing the threshold concepts, most students were unfamiliar with it. They acknowledged that their lack of understanding of threshold concepts hindered their analytical abilities and clinical decision-making, making it challenging to handle complex and varied clinical scenarios. Consequently, many students felt insufficiently confident in their future capabilities to diagnose and treat patients.

This study conducted research through semi-structured interviews with teachers and open-text surveys with students. The research team initially conducted in-depth interviews with clinical teachers, focusing on four core dimensions: clinical thinking, skill operations, humanistic spirit, and teamwork. Concurrently, open-ended free-text questionnaires were administered to medical students. After the data collection phase, the research team organized focus group discussions with expert teachers, who unanimously agreed to focus on the key bottlenecks in students’ understanding: clinical reasoning, therapeutic risk-taking, and physician-patient relationships.

### Threshold concept 1: clinical reasoning

The Paradigm Shift from Knowledge Integration to Diagnostic Thinking. The essence of clinical reasoning lies in the cognitive leap from fragmented knowledge to systematic thinking. Although medical students can memorize various disease characteristics, they still face significant challenges in dynamically integrating patient history, physical examination, and laboratory results to form diagnostic hypotheses. This cognitive gap is particularly evident in the diagnostic practice of complex diseases such as systemic lupus erythematosus (SLE). As a classic diffuse connective tissue disease, the clinical diagnosis of SLE has three specificities: First, its clinical manifestations exhibit multisystem heterogeneity, involving nine organ systems including hematologic, musculoskeletal, and neuropsychiatric systems (12); second, it lacks specific biomarkers, although antinuclear antibodies (ANA) have screening value, about 5% of confirmed patients may present ANA-negative (13); third, the disease course has dynamic evolution characteristics, requiring continuous clinical observation for diagnostic optimization (14). The linear diagnostic model in traditional medical education shows significant limitations in SLE teaching practice.

The cognitive breakthrough exhibited by the first group of students through Threshold Concepts training revealed key pathways for reconstructing clinical thinking: (1) Transformative Breakthrough in Diagnostic Paradigm: From mechanical application of the ‘11 classification criteria’ to establishing a probabilistic diagnostic framework: Students gradually mastered weighted analysis (e.g., diagnostic weight of the lupus band test > oral ulcers) and exclusionary diagnosis (e.g., differentiation from rheumatoid arthritis), forming dynamic diagnostic thinking. (2) Integrative Connection of Multidimensional Evidence: Establishing a three-dimensional linkage model of ‘immune mechanisms-clinical manifestations-laboratory evidence’: For example, anti-dsDNA antibodies not only serve as diagnostic markers, but their titer changes also reflect the extent of renal involvement; dynamic monitoring of complement levels can assist in assessing disease activity. (3) Decision-Making Breakthrough for Atypical Cases: In cases where diagnostic criteria are insufficient, students developed systematic evaluation strategies: First confirming multisystem involvement patterns (e.g., concurrent photosensitivity and thrombocytopenia), then seeking alternative evidence (e.g., direct immunofluorescence on skin biopsy), and finally establishing diagnostic verification pathways (evaluating response to experimental hormone therapy).

This cognitive transformation has irreversible characteristics, manifesting in three major clinical practice shifts: From simple quantitative diagnostic items to systematic pattern recognition; from single laboratory index interpretation to constructing multidimensional evidence chains; from binary judgment to probabilistic clinical decision-making. The study shows that students trained in threshold concepts increased SLE diagnostic accuracy by 37%, particularly excelling in identifying atypical cases.

This teaching practice suggests that modern medical education needs to transcend traditional knowledge transmission models by constructing a three-stage training system of ‘cognitive conflict-concept deconstruction-clinical reconstruction,’ helping medical students transition from standard executors to clinical decision-makers. This clinical thinking training centered on threshold concepts provides a new paradigm for cultivating clinicians with dynamic diagnostic capabilities.

### Threshold concept 2: physician-patient relationship

In recent years, the physician-patient relationship in China has been deteriorating (15–17). Studies have shown that these disputes are largely due to poor communication between doctors and patients (18). Both medical staff and patients recognize significant issues in this communication (19). The physician-patient relationship is vital to healthcare systems and medical ethics, encompassing considerations of morality, philosophy, psychology, and sociology (20). Resident physician, as direct providers of medical services and sources of reliable health information, play a crucial role. Thus, their relationship with patients affects the quality of medical services experienced by patients. Family members are also important stakeholders as they often act as primary supporters and decision-makers, especially when patients are unable to cooperate with doctors. Therefore, the relationship between doctors and patients’ family members also plays a key role in treatment and caregiving experiences.

This study found that medical students perceive the process of doctor-patient communication to be challenging. They often do not know how to begin when collecting medical histories and are unable to systematically complete this task. Some students fear that families may suspect an invasion of patient privacy, while others worry about triggering conflicts. These concerns prevent students from adequately collecting medical histories, thus hindering their ability to promptly make preliminary diagnoses. Educators emphasize the importance of reducing self-focus, directing attention toward patients, and overcoming the fear of offending or causing discomfort to them. This study involves fourth-year undergraduate medical students who have not yet begun their clinical internships and lack experience with doctor-patient interactions. Consequently, when facing standardized patients, they tend to become more nervous, affecting their diagnostic accuracy.

To help medical students grasp the threshold concept of “physician-patient relationship,” students need to overcome discomfort in communication. They should collect medical histories through exploration, collaboration, and literature review, actively seeking information on patients’ diseases, social, and psychological issues. Additionally, medical students can engage in more clinical role-playing scenarios as part of clinical situational teaching. Studies have shown that clinical situational teaching can be an effective method for teaching physician-patient relationships and clinical communication skills, enhancing medical students’ confidence and understanding of various clinical communication tasks, thus establishing good physician-patient relationships (21). Our Teaching Strategies: (1) bidirectional dialog: encourage medical students to realize that the goal of communication is not just to convey information but to establish a therapeutic alliance. (2) From “Language Skills” to “Non-Verbal Empathy”: Train students to keenly observe patients’ micro-expressions and body movements, recognize their emotions, and adjust their own body language accordingly. (3) Role-Playing as Patients: Encourage students to put themselves in the patient’s shoes, considering what the patient wants. For instance, during doctor-patient communication, students should actively ask, “What are you most worried about?” rather than simply listing treatment options.

Through our threshold concepts training, medical students have an epiphany that the doctor-patient relationship should shift from “task-based dialog” to “empathetic connection.” (1) Transformative: From “fulfilling the duty to inform” to “shared decision-making.” In case-based teaching and open-text interviews, students realize that communication is not just a skill but a therapeutic tool. The goal of communication is to establish trust between doctor and patient and to make shared decisions. (2) Irreversible: Once the “centrality of the patient perspective” is understood, it is impossible to revert to a “doctor-centered” model. (3) Integrative: The communication process must integrate medical knowledge, ethical principles, cultural context, and emotional support to form a systematic dialog framework. Before the training, about 80% of the sentences used by medical students in doctor-patient communication were medical explanations; after the training, about 65% were emotional responses and open-ended questions. (4) Challenging: It requires facing patients’ emotions (such as anger and despair) rather than avoiding them. The realization of the threshold in doctor-patient communication ultimately leads students to understand that good communication allows doctors to obtain more effective clinical information, thus better diagnosing and treating diseases. It encapsulates the essence of “Sometimes to cure, often to help, always to comfort.”

### Threshold concept 3: therapeutic risk-taking

The concept of treatment risk-taking emerged from the students’ free-text interviews as a threshold concept. In our study, we found that students felt confused and uneasy when making clinical decisions involving treatment risks. One multiple-choice question on treatment risk asked whether analgesic treatment should be given to a patient with abdominal pain of unknown etiology.

We analyzed this through the four characteristics of threshold concepts: (1) Transformative: Students achieved a cognitive shift from “risk avoidance” to “risk management.” Traditional medical education often pursues the ideal state of “zero risk,” while threshold concept teaching uses clinical cases such as acute abdominal pain analgesia to guide students in facing the inevitable risks in medical practice (e.g., analgesia possibly masking the condition), thereby deeply understanding the core concept that “risk minimization ≠ risk elimination.” This cognitive shift was reflected in students’ reflection logs: “I gradually realized that a doctor’s duty is not to eliminate all risks but to weigh the pros and cons and choose the most clinically valuable risk-taking plan.” (2) Irreversible: Students completed the reconstruction of clinical decision-making thinking. Once they grasped the dynamic balance principle of treatment risk-taking (e.g., the balance point between the timing of analgesia and diagnostic safety), students could no longer revert to the simplistic “black-and-white” decision model. Follow-up assessments showed that 75% of experimental group students in similar clinical situations could proactively suggest: “It is necessary to comprehensively evaluate the patient’s pain level and stability of vital signs before deciding whether to implement analgesic treatment.” (3) Integrative: Teaching promoted the organic integration of multidimensional knowledge. Students needed to systematically integrate multiple elements such as clinical guidelines, patients’ subjective feelings, and healthcare system resources to form a comprehensive risk assessment framework. (4) Bounded: Clarified standards for the division of medical responsibility. Through simulated medical dispute scenarios (e.g., family members questioning whether analgesia led to misdiagnosis), students learned to clearly communicate risk boundaries (e.g., “Vital signs still need to be re-evaluated every 2 h after analgesia”) and reasonably define doctor-patient responsibilities (e.g., “Doctors are responsible for providing treatment plans, and patients and families are responsible for making choices”). (5) Through the systematic application of threshold concepts in teaching, students not only mastered the method of risk quantification but more importantly internalized the professional spirit of “shared risk-taking.” This transformation strongly validates that threshold concept teaching can transcend surface-level knowledge transmission and directly address the essential goal of medical education—cultivating clinical decision-makers who possess the courage to undertake uncertainty. This teaching approach effectively bridges the gap between theoretical teaching and clinical practice, providing new insights for training the next generation of medical professionals.

In traditional medical education, students typically assume the role of passive recipients, receiving directives from teachers about what is right and wrong. Western principles of teaching and patient interaction emphasize patient autonomy and focus on patient-centered care. In this context, active student participation, experiential learning, feedback, and practice take precedence. This experiential, skills-based learning approach better aligns with the threshold concept, facilitating deeper understanding and insight (22–24).

This scholar believes that guiding students to continuously ask questions and refine their clinical diagnostic thinking helps them identify differences and similarities in problems they encounter. By leveraging their existing knowledge, students can address new challenges. When their current knowledge falls short, the process of assimilation and accommodation will foster interactions between clinical practice, foundational knowledge, and clinical reasoning. This generates new insights, consolidates learning, and ultimately enhances their understanding, enabling them to solve clinical questions effectively.

In the context of medical education, threshold concepts have been identified as crucial in shaping the professional identity and clinical reasoning skills of medical students. For instance, a scoping review of threshold concepts in medical education highlights their transformative nature, which is essential for designing curricula that span the entire medical education continuum (1). Similarly, the analysis of longitudinal integrated clerkships (LIC) in medical education reveals that these placements provide transformative learning experiences that align with the characteristics of threshold concepts, such as professional identity formation and comfort with uncertainty (25). These findings suggest that integrating threshold concepts into medical curricula can significantly enhance the learning experience by fostering critical thinking and adaptability among students.

The exploration of threshold concepts extends beyond medical education into fields such as pharmacy and occupational therapy. In pharmacy education, threshold concepts have been identified as key to understanding the Pharmacists’ Patient Care Process (PPCP), aiding in the transition from student to practitioner by making implicit aspects of patient care more explicit (26). In occupational therapy, the identification of threshold concepts through the Delphi technique has provided valuable insights into the competencies required for professional practice, thereby informing curriculum development and teaching strategies (27). These examples illustrate the broad applicability of threshold concepts in facilitating the transformation of students into competent professionals across various disciplines.

### Limitations

This study has several limitations. First, the sample size was relatively small and drawn from a single institution, which may limit the generalizability of the findings. Future multi-institutional studies with larger sample sizes are warranted. Second, although a crossover design was used to allow all participants to receive both interventions and increase statistical efficiency, potential carry-over effects cannot be entirely ruled out. Despite utilizing two distinct cases and separating the sessions by a sufficient time interval, familiarity with the simulation environment might have influenced performance in the second case. A parallel-group design would be needed to conclusively eliminate this concern.

## Conclusion

This study utilized medical scenario simulations to train undergraduate clinical medicine students using two different methods. Participants reached a consensus on three threshold concepts vital for clinical undergraduate education: clinical reasoning, therapeutic risk-taking, and the physician-patient relationship. Results indicate that students trained with a focus on threshold concepts show an enhanced ability to manage and learn clinical medical problems. These concepts can potentially inform the development of competencies in disease diagnosis and therapy, guiding teaching and learning activities to facilitate the transformation into competent physicians. In conclusion, the concept of threshold concepts, as articulated by Meyer and Land, offers a powerful framework for understanding and facilitating transformative learning across various disciplines. By identifying and integrating these concepts into educational curricula, educators can enhance the learning experience, promote professional identity formation, and equip students with the critical thinking skills necessary for success in their respective fields. The continued exploration and application of threshold concepts hold significant promise for advancing educational practices and improving student outcomes.

## Data Availability

The original contributions presented in the study are included in the article/supplementary material, further inquiries can be directed to the corresponding authors.
